# Altered Fatty Acid Metabolism-Related Gene Expression in Liver from Morbidly Obese Women with Non-Alcoholic Fatty Liver Disease

**DOI:** 10.3390/ijms151222173

**Published:** 2014-12-02

**Authors:** Teresa Auguet, Alba Berlanga, Esther Guiu-Jurado, Salomé Martinez, José Antonio Porras, Gemma Aragonès, Fátima Sabench, Mercé Hernandez, Carmen Aguilar, Joan Josep Sirvent, Daniel Del Castillo, Cristóbal Richart

**Affiliations:** 1 Grup de Recerca GEMMAIR (AGAUR)-Medicina Aplicada, Departament de Medicina i Cirurgia, Universitat Rovira i Virgili (URV), Institut d’Investigació Sanitària Pere Virgili IISPV (IISPV), Tarragona 43003, Spain; E-Mails: tauguet.hj23.ics@gencat.cat (T.A.); alba.berlanga@urv.cat (A.B.); esther.guiu@urv.cat (E.G.-J.); gemma.aragones@iispv.cat (G.A.); caguilar.hj23.ics@gencat (C.A.); 2 Servei Medicina Interna, Hospital Universitari Joan XXIII Tarragona, Mallafré Guasch, 4, Tarragona 43007, Spain; E-Mail: aporras.hj23.ics@gencat.cat; 3 Servei Anatomia Patològica, Hospital Universitari Joan XXIII Tarragona, Mallafré Guasch, 4, Tarragona 43007, Spain; E-Mails: mgonzalez.hj23.ics@gencat.cat (S.M.); jsirvent.hj23.ics@gencat.cat (J.J.S.); 4 Servei de Cirurgia, Hospital Sant Joan de Reus, Departament de Medicina i Cirurgia, Universitat Rovira i Virgili (URV), IISPV, Avinguda Doctor Josep Laporte, 2, Tarragona 43204, Spain; E-Mails: fatima.sabench@urv.cat (F.S.); mhernandezg@grupsagessa.com (M.H.); ddelcastillo@grupsagessa.com (D.D.C.)

**Keywords:** insulin resistance, morbid obesity, fatty acid metabolism, non-alcoholic fatty liver disease, simple steatosis, non-alcoholic steatohepatitis

## Abstract

Lipid accumulation in the human liver seems to be a crucial mechanism in the pathogenesis and the progression of non-alcoholic fatty liver disease (NAFLD). We aimed to evaluate gene expression of different fatty acid (FA) metabolism-related genes in morbidly obese (MO) women with NAFLD. Liver expression of key genes related to *de novo* FA synthesis (LXRα, SREBP1c, ACC1, FAS), FA uptake and transport (PPARγ, CD36, FABP4), FA oxidation (PPARα), and inflammation (IL6, TNFα, CRP, PPARδ) were assessed by RT-qPCR in 127 MO women with normal liver histology (NL, *n* = 13), simple steatosis (SS, *n* = 47) and non-alcoholic steatohepatitis (NASH, *n* = 67). Liver FAS mRNA expression was significantly higher in MO NAFLD women with both SS and NASH compared to those with NL (*p* = 0.003, *p* = 0.010, respectively). Hepatic IL6 and TNFα mRNA expression was higher in NASH than in SS subjects (*p* = 0.033, *p* = 0.050, respectively). Interestingly, LXRα, ACC1 and FAS expression had an inverse relation with the grade of steatosis. These results were confirmed by western blot analysis. In conclusion, our results indicate that lipogenesis seems to be downregulated in advanced stages of SS, suggesting that, in this type of extreme obesity, the deregulation of the lipogenic pathway might be associated with the severity of steatosis.

## 1. Introduction

Non-alcoholic fatty liver disease is characterized by an accumulation of triglycerides (TG) in hepatocytes and has frequently been associated with obesity, type 2 diabetes mellitus, hyperlipidemia, and insulin resistance (IR) [1]. NAFLD is an increasingly recognized condition associated with increased cardiovascular and liver-related mortality [2,3]. The pathogenesis of NAFLD has been interpreted by the “double-hit” hypothesis, comprising lipid accumulation as the primary insult or “first hit” in the liver [4,5], followed by a “second hit” in which proinflammatory mediators induce inflammation, hepatocellular injury and fibrosis [6]. Recently, however, some studies have shown that while hepatic TG accumulation seems to be a benign symptom of hepatic steatosis, fatty acid (FA) metabolites contribute to the progression of NAFLD to NASH. IR promotes the recruitment of free FAs from the serum pool as well as intrahepatic fatty acid accumulation, which induces apoptosis and the formation of reactive oxygen species (ROS). FAs themselves also promote hepatic insulin resistance via Toll-like receptor 4 (TLR4) activation that increases the release of inflammatory biomediators such as IL6, IL1β, and the TNFα receptor [7], indicating a vicious cycle of lipid accumulation, and IR as a crucial mechanism in the pathogenesis of NASH, among other mechanisms. In this regard, some authors have suggested a “multiple parallel hits hypothesis” to explain the pathophysiology of NAFLD [8,9].

Lipid accumulation in the human liver seems to be a crucial mechanism in NAFLD pathophysiology, so its regulatory mechanisms need to be understood in order to control the progression of NAFLD. It is known that hyperinsulinemia promotes *de novo* synthesis of fatty acids from glucose and increases free FA flux to the liver due to peripheral IR through the sterol regulatory element-binding protein-1c (SREBP1c) and inhibits fatty acid oxidation through the nuclear receptor peroxisome proliferators-activated receptor-α (PPARα). Then, insulin signalling and nuclear receptors (including PPARs and LXRα) regulate both hepatic fatty acid and glucose metabolism. Both pathways are closely interrelated and share common regulatory elements and indistinguishably contribute to NAFLD [10]. Regarding that, some authors have described overexpression of genes involved in FA partitioning and binding, lipolysis and inflammation in the human fatty liver [11,12,13,14,15]. In addition, more recently, Ahn *et al.* found that LXRα expression correlated with the degree of hepatic fat deposition, as well as with hepatic inflammation and fibrosis in NAFLD patients [16].

In a previous study we demonstrated that lipogenesis and FA oxidation were downregulated in subcutaneous adipose tissue (SAT) samples from morbidly obese women, suggesting that SAT works to limit any further development of fat mass [17]. Based on that data, we wished to further investigate the fatty acid metabolism in the liver of MO women with NAFLD by evaluating the expression of some key genes involved in *de novo* synthesis of fatty acids (LXRα, SREBP1c, ACC1, FAS), fatty acid uptake and transport (PPARγ, CD36, FABP4), fatty acid oxidation (PPARα) and, finally, inflammation related genes (IL6, TNFα, CRP, PPARδ). Furthermore, as the lipid accumulation in the cytoplasm of hepatocytes seems to be the hallmark of NAFLD, we assessed the relationship between the expression of these genes and the presence of hepatic fat accumulation in this cohort.

## 2. Results

### 2.1. Baseline Characteristics of Subjects

The cohort of morbidly obese women was classified according to the liver pathology into normal liver (NL), simple steatosis (SS) and non-alcoholic steatohepatitis (NASH) (Table 1). Age and anthropometrical measurements were not significantly different between the three morbidly obese groups. However, insulin and HbA1c levels were significantly higher in both SS and NASH groups than in the NL group. Glucose levels were significantly higher in SS and tended to be higher in NASH (*p* = 0.05), compared with the NL group. Also, IL6 levels were significantly higher in NASH than in the NL group. Our results indicated that ALT and ALP activity was higher in both SS and NASH groups than in obese women with normal liver histology. Furthermore, AST levels tended to be higher in SS (*p* = 0.05) and were significantly higher in NASH, compared with the NL group.

**Table 1:** Anthropometric and metabolic variables of the study cohort classified according to the liver pathology.

Variables	NL (*n* = 13)	SS (*n* = 47)		NASH (*n* = 67)		
	Mean ± SEM	Mean ± SEM	*p*-Value 1	Mean ± SEM	*p*-Value 1	*p*-Value 2
Age (years)	44.5 ± 3.2	47.7 ± 1.5	n.s	47.1 ± 1.3	n.s	n.s
Weight (kg)	122.9 ± 4.3	121.6 ± 2.4	n.s	119.4 ± 1.8	n.s	n.s
WC (cm)	131.5 ± 6.2	128.7 ± 1.6	n.s	130.8 ± 1.7	n.s	n.s
BMI (kg/m^2^)	49.1 ± 1.9	48.3 ± 1.1	n.s	46.5 ± 0.5	n.s	n.s
Glucose (mg/dL)	100.8 ± 6.8	128.1 ± 6.2	0.026	128.8 ± 6.0	0.05	n.s
Insulin (mUI/L)	13.7 ± 2.6	20.4 ± 1.7	0.048	23.4 ± 3.1	0.04	n.s
HbA1c (%)	5.1 ± 0.3	6 ± 0.3	0.031	6.3 ± 0.2	0.028	n.s
HOMA2-IR	2.1 ± 0.5	2.7 ± 0.2	n.s	2.9 ± 0.5	n.s	n.s
HDL-C (mg/dL)	43.3 ± 2.6	39.4 ± 1.8	n.s	39.6 ± 1	n.s	n.s
LDL-C (mg/dL)	96 ± 6.9	99.7 ± 4.3	n.s	100.7 ± 3.7	n.s	n.s
Triglycerides (mg/dL)	142.7 ± 13	197.2 ± 15	n.s	156.6 ± 7.9	n.s	n.s
AST (U/L)	25.6 ± 4	45.6 ± 5.3	0.05	43.2 ± 3.5	0.042	n.s
ALT (U/L)	24.6 ± 2.4	44.3 ± 4.6	<0.001	43.2 ± 3.3	<0.001	n.s
GGT (U/L)	23.3 ± 6.9	28.6 ± 3.2	n.s	36.2 ± 5.2	n.s	n.s
ALP (U/L)	57.8 ± 3.2	68.3 ± 2.4	0.028	71.4 ± 2.6	0.032	n.s
**Adipo/Cytokine Circulating Levels**						
HMW adiponectin (µg/mL)	3.8 ± 1.7	3.3 ± 0.7	n.s	3 ± 0.4	n.s	n.s
IL6 (pg/mL)	2.1 ± 0.3	2.7 ± 0.5	n.s	3.3 ± 0.5	0.031	n.s
TNFRI (ng/mL)	2.8 ± 0.3	3.1 ± 0.2	n.s	3 ± 0.2	n.s	n.s
TNFRII ng/mL	4.2 ± 0.7	5.2 ± 0.5	n.s	5.7 ± 0.4	n.s	n.s
CRP (mg/dL)	2.2 ± 2.1	1.4 ± 0.3	n.s	3 ± 0.8	n.s	0.046
FABP4 (ng/mL)	56.8 ± 16.9	62.6 ± 4.6	n.s	56.4 ± 5.1	n.s	n.s

NL, morbidly obese subjects with normal liver; SS, morbidly obese subjects with simple steatosis; NASH, morbidly obese subjects with steatohepatitis; ALT, alanine aminotransferase; ALP, alkaline phosphatase; AST, aspartate aminotransferase; BMI, body mass index; CRP, C reactive protein; FABP4, fatty acid binding protein 4; GGT, gamma-glutamyltransferase; HbA1c, glycosylated haemoglobin; HDL-C, high density lipoprotein cholesterol; HOMA2-IR, homeostatic model assessment 2-insulin resistance; IL6, interleukin 6; LDL-C, low density lipoprotein cholesterol; TNFRI and II, tumour necrosis factor receptor I and II; WC, waist circumference. ANOVA test was used to compare the gene expression in the different groups. *p*-Value 1 indicates significant differences respect NL group (*p* < 0.05); *p*-Value 2 indicates significant differences respect SS group (*p* < 0.05). n.s indicates no significant differences. Data are expressed as mean ± SEM.

### 2.2. Evaluation of the Expression of Genes Related to Lipid Metabolism and Inflammation in Liver and Their Protein Expression

We analysed liver expression, in our cohort of morbidly obese women, of some key genes related to the *de novo* synthesis of fatty acids (LXRα, SREBP1c, ACC1, FAS), fatty acid (FA) uptake and transport (PPARγ, CD36, FABP4), FA oxidation (PPARα), and related to inflammation (IL6, TNFα, CRP, PPARδ).

We first classified the whole cohort into NL, SS, and NASH (Table 2). The results indicate that among the key genes related to the *novo* fatty acid synthesis, only FAS mRNA expression was significantly higher in MO NAFLD women with both SS and NASH compared to those with normal liver histology. Regarding inflammation, IL6 hepatic mRNA expression was significantly higher in NASH than in the SS group. Hepatic TNFα mRNA expression tended to be higher in NASH compared with the SS group (*p* = 0.05). No more significant differences were found regarding the other studied fatty acid metabolism-related genes (Table 2).

**Table 2:** Hepatic expression of genes related to de *novo* fatty acid synthesis, fatty acid uptake and transport, fatty acid oxidation, and inflammation in morbidly obese women according to the liver pathology.

Gene Expression	NL ( *n* = 13)	SS (*n* = 47)		NASH (*n* = 67)		
	Mean ± SEM	Mean ± SEM	*p*-Value 1	Mean ± SEM	*p*-Value 1	*p*-Value 2
***De novo* lipogenesis**						
LXRα	9.5 ± 2.6	10.4 ± 1.4	n.s	8.5 ± 1.1	n.s	n.s
SREBP1c	8.1 ± 1.3	10.2 ± 1	n.s	8.7 ± 0.9	n.s	n.s
ACC1	4.4 ± 1.0	6.7 ± 1.7	n.s	7.6 ± 2.1	n.s	n.s
FAS	5.9 ± 1.1	13.9 ± 2.3	0.003	16.8 ± 2.8	0.001	n.s
**Fatty acid uptake and transport**						
PPARγ	2.9 ± 0.5	4.6 ± 0.7	n.s	4.8 ± 1.1	n.s	n.s
CD36	6.1 ± 0.9	6.3 ± 0.7	n.s	5.8 ± 0.7	n.s	n.s
FABP4	1.1 ± 0.4	3.3 ± 0.8	n.s	3.5 ± 1.3	n.s	n.s
**Fatty acid oxidation**						
PPARα	26.1 ± 4.5	26.6 ± 3.6	n.s	21.2 ± 3	n.s	n.s
**Inflammation**						
IL6	1.1 ± 0.7	0.5 ± 0.1	n.s	1.5 ± 0.4	n.s	0.033
TNFα	0.8 ± 0.6	0.4 ± 0.1	n.s	1 ± 0.2	n.s	0.050
CRP	117.4 ± 19.9	167.4 ± 30	n.s	165.4 ± 26.4	n.s	n.s
PPARδ	3.6 ± 0.8	4.9 ± 0.7	n.s	3.7 ± 0.5	n.s	n.s

NL, morbidly obese subjects with normal liver; SS, morbidly obese subjects with simple steatosis; NASH, morbidly obese subjects with steatohepatitis. ANOVA test was used to compare the gene expression in the different groups. *p*-Value 1 indicates significant differences respect NL group (*p* < 0.05); *p*-Value 2 indicates significant differences respect SS group (*p* < 0.05). n.s indicates no significant differences. Data are expressed as mean ± SEM.

Then, in order to add to the current knowledge about the role of lipid metabolism alterations in simple steatosis, we assessed the relationship between the expression of the studied genes and the presence of hepatic fat accumulation, classifying the SS group into different grades: mild, moderate or severe SS (Table 3).

**Table 3:** Hepatic expression of genes related to *de novo* fatty acid synthesis, fatty acid uptake and transport, fatty acid oxidation, and inflammation in morbidly obese women diagnosed with different degrees of simple steatosis (SS).

Gene Expression	MILD SS (*n* = 18)	MODERATE SS (*n* = 16)		SEVERE SS (*n* = 13)		
	Mean ± SEM	Mean ± SEM	*p*-Value 1	Mean ± SEM	*p*-Value 1	*p*-Value 2
***De novo* lipogenesis**						
LXRα	12.5 ± 3.1	11 ± 1.8	n.s	4.8 ± 2.2	0.05	0.05	
SREBP1c	10.9 ± 2.3	11.8 ± 1.6	n.s	8.7 ± 2.0	n.s	n.s	
ACC1	6.6 ± 1.9	6.4 ± 1.3	n.s	2.4 ± 0.4	0.042	0.008	
FAS	15.2 ± 4.4	16.3 ± 3.8	n.s	7.4 ± 1.6	n.s	0.047	
**Fatty acid uptake and transport**						
PPARγ	6.1 ± 1.6	4.7 ± 1.2	n.s	2.9 ± 0.9	n.s	n.s
CD36	5.4 ± 1.4	8.4 ± 0.9	n.s	5.3 ± 1.2	n.s	n.s
FABP4	1.7 ± 0.6	4.7 ± 1.4	n.s	4.9 ± 2.3	n.s	n.s
**Fatty acid oxidation**													
PPARα	31.5 ± 7.9	24.5 ± 4.9	n.s	23.1 ± 6.2	n.s	n.s
**Inflammation**						
IL6	0.6 ± 0.2	0.5 ± 0.1	n.s	0.4 ± 0.2	n.s	n.s
TNFα	0.3 ± 0.1	0.4 ± 0.1	n.s	0.5 ± 0.2	n.s	n.s
CRP	129.9 ± 63.7	132.7 ± 34.5	n.s	139 ± 64.1	n.s	n.s
PPARδ	5.7 ± 1.2	5.6 ± 1	n.s	4.3 ± 2.2	n.s	n.s

NL, morbidly obese subjects with normal liver; MILD SS, morbidly obese subjects with mild simple steatosis; MODERATE SS, morbidly obese subjects with moderate simple steatosis; SEVERE SS, morbidly obese subjects with severe simple steatosis. ANOVA test was used to compare the gene expression in the different groups. *p*-Value 1 indicates significant differences respect MILD SS group (*p* < 0.05); *p*-Value 2 indicates significant differences respect MODERATE SS group (*p* < 0.05). n.s indicates no significant differences. Data are expressed as mean ± SEM.

Regarding *de novo* synthesis of fatty acids, liver ACC1 mRNA expression was down regulated in severe SS compared to both mild and moderate SS groups. In addition, LXRα mRNA expression tended to be lower in severe SS compared to both mild and moderate SS groups (*p* = 0.05). Hepatic FAS mRNA expression levels were significantly lower in severe SS compared to the moderate SS group. Regarding SREBP1c, despite we did not find any significant difference, its mRNA expression seem to be lower in severe SS compared to both mild and moderate SS groups (Table 3).

**Figure 1:**
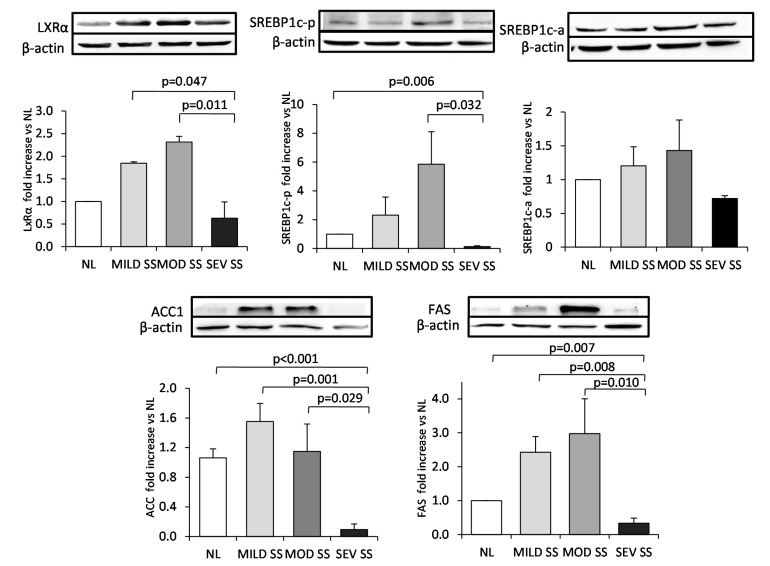
Liver expression of lipogenic transcription factors and enzymes in morbidly obese patients diagnosed with different degree of simple steatosis. Representative Western blot analysis showing LXRα, SREBP1c-precursor form (SREBP1c-p), SREBP1c-active form (SREBP1c-a), ACC1, FAS and β-actin protein expression and bar graphs showing the quantification of bands normalized by values of β-actin bands (*n* = 28: 6 NL, 8 MILD SS, 8 MOD SS, 6 SEV SS). Results are shown as mean ± SD. *p* < 0.05 are considered statistically significant. NL, morbidly obese subjects with normal liver; MILD SS, morbidly obese subjects with mild simple steatosis; MOD SS, morbidly obese subjects with moderate simple steatosis; SEV SS, morbidly obese subjects with severe simple steatosis.

In order to confirm these results regarding lipogenic gene expression, we also analyzed the protein expression of the key genes related to the *de novo* fatty acid synthesis by western blot analysis. There were similar results with respect to its protein expression and those obtained in the gene expression analysis. LXRα, SREBP1c precursor form, ACC1 and FAS protein expression was significantly lower in morbidly obese women with severe SS compared to those with moderate SS (*p* < 0.05). We also determined the activated SREBP1c form by Western blot analysis. Our results show that, although we did not find any significant difference between groups, activated SREBP1c protein has a similar profile of both ACC1 and FAS mRNA and protein expression, two well known target genes of SREBP1c (Figure 1).

We also studied the genes related to FA oxidation, FA uptake and transport, and related to inflammation in the SS cohort classified into different grades of simple steatosis. In this case, we did not find any significant difference on its mRNA expression levels (Table 3).

### 2.3. Correlations between the Expression of Genes Related to Lipid Metabolism and Inflammation with Glucose Metabolism Parameters

Our results showed that liver FAS expression correlated positively with glucose circulating levels in the morbidly obese cohort (Table 4).

Regarding FA uptake and transport, hepatic CD36 expression correlates positively with insulin circulating levels, and also with HOMA2-IR in the morbidly obese group. In addition, we found a positive correlation between FABP4 expression and glucose, insulin, HbA1c and HOMA2-IR (Table 4). Interestingly, when we classify the morbidly obese cohort into NL, SS and NASH, we observed that both CD36 and FABP4 correlations with glucose metabolism parameters became stronger in NASH (CD36: Insulin *r* = 0.550, *p* = 0.010; HOMA2-IR *r* = 0.546, *p* = 0.010) (FABP4: Glucose *r* = 0.801, *p* < 0.001; Insulin *r* = 0.833, *p* < 0.001; HbA1c *r* = 0.893, *p* < 0.001; HOMA2-IR *r* = 0.838, *p* < 0.001).

Finally, liver IL6 expression correlated positively with insulin circulating levels and with HOMA2-IR in the morbidly obese group (Table 4).

**Table 4:** Significant correlations between the expression of genes related to lipid metabolism and inflammation with glucose metabolism parameters in the morbidly obese cohort.

Variables	FABP4		FAS		CD36		IL6	
	*r*	*p*-Value	*r*	*p*-Value	*r*	*p*-Value	*r*	*p*-Value
Glucose (mg/dL)	0.465	0.001	0.185	0.035	−0.055	0.697	0.014	0.925
Insulin (mu/L)	0.710	<0.001	0.008	0.933	0.357	0.013	0.371	0.011
Homa2-IR	0.714	<0.001	0.079	0.514	0.354	0.014	0.369	0.012
Hba1c (%)	0.742	<0.001	0.118	0.24	0.155	0.325	0.185	0.252

HbA1c, glycosylated haemoglobin; HOMA2-IR, homeostatic model assessment 2-insulin resistance.

## 3. Discussion

The novelty of this study lies in the fact that it establishes a clear relationships between NAFLD and genes related to *de novo* synthesis of fatty acids, FA uptake and transport, FA oxidation, and related to inflammation, in an extensive cohort of women with morbid obesity (BMI > 40 kg/m^2^). Moreover, this design provides a comparison of gene expression and protein levels between different degrees of simple steatosis according to the percentage of liver fat deposition (mild, moderate or severe SS).

The present study demonstrates that FAS, well known as an important lipogenic enzyme, is overexpressed in the liver of MO NAFLD patients with both simple steatosis and non-alcoholic steatohepatitis, in agreement with other authors [13,14,18]. Other studies have shown enhanced expression of LXRα and SREBP1c in NAFLD [13,14,15,18,19]. However, we were unable to find any other significant difference in other related genes to the *de novo* lipogenesis pathway. These discrepancies might be explained by differences in the cohort of the patients studied. Higuchi *et al.* [13] included normal weight patients with NAFLD and Lima-Cabello *et al.* [14] included patients with NAFLD and with steatosis related to chronic hepatitis C virus infection in mildly overweight men and women. Finally, Nakamuta *et al.*, who included a cohort of obese patients, observed that the expression of LXRα and ACC1 was upregulated in NAFLD and it was more noticeable in non-obese than in obese patients [19].

In order to add to the current knowledge about the role of lipid metabolism alterations in simple steatosis, we evaluated the expression of these genes in morbidly obese patients with different histopathology types of SS according to the hepatic fat deposition. The most outstanding finding of the present study is that the liver expression of key genes related to *de novo* fatty acid synthesis (LXRα, ACC1 and FAS) have an inverse relationship with the grade of steatosis, that is to say, it diminishes when the grade of steatosis increases. This novel finding was confirmed evaluating the protein expression, obtaining similar results with respect to LXRα, ACC1, FAS levels, and also with respect to SREBP1c. Our findings indicate that, in this type of extreme obesity, the hepatic lipogenic pathway seems to be downregulated in advanced stages of simple steatosis. The explanation for these results are complex, however experimental studies have shown that in mice with total insulin resistance in liver, insulin fails to stimulate the synthesis of fatty acids and triglycerides [20,21]. In this context, the liver of an extremely obese patient with severe steatosis might behave as if there were total insulin resistance, which could be responsible for the downregulation of the lipogenic pathway shown in the liver of these patients. However, in the present study we did not perform hyperinsulinemic euglycemic clamp to measure hepatic insulin sensitivity in order to confirm this hypothesis.

We also found that IL6 and TNFα, two important proinflammatory adipocytokines hugely expressed in the adipose tissue of obese human subjects and patients with IR [22,23], were overexpressed in the liver of NAFLD MO women with NASH compared to those with simple steatosis. These results are in agreement with the literature, which supports that they correlate with histological severity in obese patients. For instance, Crespo *et al.* reported increased hepatic expression of TNFα in patients with NASH compared to SS patients [24]. Moreover, Wieckowska *et al.* demonstrated markedly increased IL6 expression in the liver of NAFLD patients with NASH compared to those with SS or normal liver [25].

Insulin resistance is known to be an intrinsic defect in NAFLD that is closely associated with steatosis, inflammation and disease progression in NASH. Moreover, IR has been described as the main factor associated with NASH development, as well as genetic and environmental factors [12,26,27]. In this regard, we found correlations between some important genes related to the hepatic uptake and transport of fatty acids (CD36 and FABP4) and the presence of IR and insulin circulating levels in our cohort of MO NAFLD women, becoming stronger in those with NASH. These findings are in agreement with Miquilena-Colina *et al.* who observed a significant correlation between hepatic CD36 expression and plasma insulin levels and insulin resistance (HOMA-IR) in patients with NASH [28]. Furthermore, we observed the same correlation regarding IL6 gene expression. These results indicate that hepatic fatty acid accumulation, as well as inflammation, might be contributing to NAFLD progression in relation with insulin resistance.

In addition, we found a positive correlation between glucose circulating levels and liver FAS expression in the MO cohort. This finding supports the reported observation that glucose binds and activates LXRs transcription factors and induces their target genes, including SREBP1c, ACC1 and FAS [29,30].

Our cohort of severely obese women has made it possible to establish clear relationships between NAFLD and fatty acid metabolism-related genes without the interference of such confounding factors as gender or age. These results cannot be extrapolated to other obesity groups, normal-weight or over-weight women or men. Further studies, including these cohorts, would be useful in order to validate our findings. Another limitation is that we did not assess the hepatic insulin sensitivity by hyperinsulinemic euglycemic clamp.

## 4. Materials and Methods

### 4.1. Subjects

The study was approved by the institutional review board (Comitè d’Ètica d’Investigació Clínic, Hospital Sant Joan de Reus, 09-06-25/6proj2). All participants gave written informed consent for participation in medical research. We included 127 morbidly obese women (BMI > 40 kg/m^2^) of Western European descent. Liver biopsies were obtained during planned laparoscopic bariatric surgery. All biopsies were performed for clinical indications. 

The diagnosis of NAFLD was made using the following criteria: (1) liver pathology; (2) an intake of less than 10 gr. of ethanol/day; and (3) appropriate exclusion of other liver diseases.

The weight of all subjects was stable with no fluctuation in body weight greater than 2% for at least 3 months prior to surgery. The exclusion criteria were: (1) concurrent use of medications known to produce hepatic steatosis; (2) patients using lipid-lowering medications including PPARα or -γ agonists; (3) diabetic women who were receiving insulin or on medication likely to influence endogenous insulin levels; (4) menopausal and post-menopausal women and subjects receiving contraceptive treatment; (5) patients who had an acute illness, current evidence of acute or chronic inflammatory or infectious diseases or end-stage malignant diseases.

### 4.2. Liver Pathology

Liver samples were stained with hematoxylin and eosin, and Manson’s trichrome stains and scored by two experienced hepatopathologists using the methods described before [31,32]. Simple steatosis (SS) was graded as follows: grade 1 or mild SS: more than 5% and less than 33% of hepatocytes affected; grade 2 or moderate SS: 33% to 66% of hepatocytes affected; or grade 3 or severe SS: more than 66% of hepatocytes affected. In addition to steatosis, the minimum criteria for the diagnosis of steatohepatitis included the presence of lobular inflammation and either ballooning cells or perisinusoidal/pericellular fibrosis in zone 3 of the hepatic acinus.

According to their liver pathology [30,31], patients were sub-classified into the following groups: (1) normal liver (NL) histology (*n* = 13); (2) simple steatosis (SS) (micro/macrovesicular steatosis without inflammation or fibrosis, *n* = 47); (3) non-alcoholic steatohepatitis (NASH) (Brunt grade 1–3, *n* = 67).

### 4.3. Biochemical Analyses

A complete anthropometrical, biochemical, and physical examination was carried out on each patient. Body height and weight were measured with the patient standing in light clothes and shoeless. Body mass index was calculated as body weight divided by height squared (kg/m^2^). The subjects’ waist circumference was measured with a soft tape midway between the lowest rib and the iliac crest. Laboratory studies included glucose, insulin, glycated haemoglobin, high-density lipoprotein cholesterol, low-density lipoprotein cholesterol, triglycerides and transaminases, all of which were analysed using a conventional automated analyser. Insulin resistance was estimated using homeostasis model assessment of IR (HOMA2-IR) [33].

We determined the circulating levels of several molecules related to inflammation including adipokines (HMW adiponectin), acute phase proteins (CRP) and proinflammatory cytokines (IL6, TNFRI and TNFRII). Circulating levels of HMW adiponectin (EMD Millipore, St. Charles, MO, USA), CRP (Dade Behring, Marburg, Germany), IL6 (Quantikine, R&D Systems, Minneapolis, MN, USA), FABP4 (Biovendor, Modrice, Czech Republic), TNFRI and TNFRII (AssayPro, St. Charles, MO, USA) were measured in duplicate using enzyme-linked immunosorbent assays (ELISA) following the manufacturer’s instructions.

### 4.4. RNA Isolation and Real-Time PCR

Liver samples were conserved in RNAlater (Sigma, Barcelona, Spain) for 24 h at 4 °C and then stored at −80 °C. Total RNA from liver was isolated according to the manufacturers’ protocols RNeasy mini kit (Qiagen, Barcelona, Spain). RNA was digested with DNase I (RNase-Free DNase set; Qiagen). First-strand cDNA was synthesized using an equal amount of total RNA with the High Capacity RNA-to-cDNA Kit (Applied Biosystems, Madrid, Spain). Real-time quantitative PCR was carried out in a final volume of 20 μL, which contained 10 ng of reverse-transcribed cDNA, 10 μL of 2× TaqMan Fast Universal PCR Master Mix (Applied Biosystems) and 1 μL TaqMan Assay predesigned by Applied Biosystems for the detection of LXRα, SREBP1c, ACC1, FAS, PPARγ, CD36, FABP4, PPARα, IL6, TNFα, CRP, PPARδ, and 18S ribosomal RNA, which was used as the housekeeping gene. The mRNA expression for each gene and sample was calculated using the recommended 2^−ΔΔ*C*t^ method. All reactions were carried out in duplicate in 96-well plates using the 7900HT Fast Real-Time PCR systems (Applied Biosystems).

### 4.5. Western Blot Analysis

Protein levels were evaluated in a subgroup of 28 subjects (6 MO women with NL, 8 with mild SS, 8 with moderate SS and 6 with severe SS), for whom enough tissue was available. Liver samples were homogenized in a medium containing 50 mM HEPES, 150 mM NaCl, 1 mM DTT, 0.1% SDS and 1% protease inhibitor cocktail (Thermo Scientific, Madrid, Spain). Protein concentrations were determined by using a BCA assay Kit (Thermo Scientific). For Western blot analysis, equal amounts of protein (50 µg) were separated by SDS/PAGE (7% acrylamide) and transferred onto nylon membranes. Non-specific binding was blocked by preincubation of the membranes with 5% (*w*/*v*) non-fat milk powder in 0.1% PBS-Tween for 1 h. Specific protein expression was detected by incubating with goat anti-LXRα (Santa Cruz Biotechnology, Inc., Heidelberg, Germany), rabbit anti-SREBP1c (Thermo Scientific), rabbit anti-ACC1 (Cell Signaling Technology, Inc., Barcelona, Spain) and rabbit anti-FAS (Cell Signaling Technology) antibodies overnight at 4 °C, followed by an incubation with anti-mouse IgG (GE Healthcare, Freiburg, Germany), anti-goat IgG (Santa Cruz Biotechnology, Inc.) or anti-rabbit IgG (GE Healthcare) antibodies for 2 h at room temperature and developed with SuperSignal West Pico Chemiluminescent or SuperSignal Femto Maximum Sensitivity Substrate (Thermo Scientific). The density of specific bands was determined by densitometry and quantified by the Phoretix 1D software from TotalLab. The expression pattern of all proteins was normalized by β-actin (Sigma) liver expression.

### 4.6. Statistical Analysis

All the values reported were analyzed using the SPSS/PC+ for windows statistical package (version 19.0; SPSS, Chicago, IL, USA). One-way ANOVA with post-hoc Tukey test was used to compare continuous variables between groups. The strength of association between variables was calculated using Pearson’s method for parametric variables and Spearman’s ρ-correlation test for non-parametric contrasts. *p-*Values <0.05 were considered to be statistically significant.

## 5. Conclusions

In conclusion, although it was not possible to determine the causality that leads to the downregulation of *de novo* fatty acid synthesis in advanced stages of simple steatosis, our results suggest that, in NAFLD patients with this type of extreme obesity, the deregulation of the lipogenic pathway might be associated with the severity of simple steatosis. Prospective studies are needed in order to better understand the alteration of fatty acid metabolism-related pathways in morbidly obese subjects with NAFLD.

## Abbreviations

ACC1: acetyl-coenzyme A carboxylase 1
ALT: alanine aminotransaminase
AST: aspartate aminotransaminase
ALP: alkaline phosphatase
BMI: body mass index
CD36: hepatic fatty acid translocase
CRP: c-reactive protein
FA: fatty acid
FABP4: fatty acid binding protein 4
FAS: fatty acid synthase
18S: 18S ribosomal RNA
GGT: γ-glutamyl transferase
HbA1c: glycosylated hemoglobin
HDL-C: high density lipoprotein cholesterol
HOMA2-IR: homeostatic model assessment method insulin resistance
IL6: interleukin 6
IL1β: interleukin 1β
IR: insulin resistance
LDL-C: low density lipoprotein cholesterol
LXRα: liver X receptor
MOD SS: moderate simple steatosis
NAFLD: non-alcoholic fatty liver disease
NASH: non-alcoholic steatohepatitis
NL: normal liver
PPARα: peroxisome-proliferator-activated receptor α
PPARδ: peroxisome-proliferator-activated receptor δ
PPARγ: peroxisome-proliferator-activated receptor *γ*
SEV SS: severe simple steatosis
SREBP1c: sterol-regulatory-element-binding protein
SS: simple steatosis
TG: triglycerides
TLR4: Toll-like receptor 4
TNFα: tumour necrosis factor
TNFRI: tumour necrosis factor receptor I
TNFRII: tumour necrosis factor receptor II
WC: waist circumference
